# A Randomized Pilot Study Comparing the Impact of Strengthening-Based Running Training with Only Running on the Incidence of Running-Related Injuries among Novice Runners

**DOI:** 10.3390/sports12010025

**Published:** 2024-01-09

**Authors:** Anh Phong Nguyen, Noé Abeels, Romain Van Brussel, Benoit Pairot de Fontenay

**Affiliations:** 1 NeuroMusculoSKeletal Laboratory (NMSK), Institut de Recherche Expérimentale et Clinique, Université Catholique de Louvain, Avenue Mounier 53, 1200 Brussels, Belgium; 2 Faculté des Sciences de la Motricité, Université Catholique de Louvain, Place Pierre de Courbertin 1, 1348 Louvain-La-Neuve, Belgium; 3 The Running Clinic, Lac Beauport, QC G3B 2J8, Canada; benoit.pdf@gmx.com; 4 Laboratoire InterUniversitaire de Biologie de la Motricité, Université de Lyon, Rue Raphael Dubois 4, 69100 Villeurbanne, France; 5 Ramsay Santé, Clinique de la Sauvegarde, 69009 Lyon, France

**Keywords:** resistance training, aerobic training, novice athletes, primary prevention, sports injuries

## Abstract

Background: Running-related injuries (RRI) are common in novice runners. Reducing early training running volume with strengthening activities may improve RRI without impeding running performance. Objectives: 1. Gather feasibility data for a randomized, controlled trial comparing a strengthening-based program to a conventional running program; 2. Assess RRI; and 3. Assess running performance. Methods: Seventy-four university students (38 females, 21 ± 2.3 years, 68.2 ± 10.8 kg, BMI: 22.6 ± 2.97), all novice runners, were randomized in two groups, i.e., a strengthening and running group (INT) and a running group (CON). The completed sessions, RRI, dropout, and maximal aerobic speed were recorded through an online application. Results: The INT group had 52.6% attrition, while the CON group had 41.7%. The INT group had 56.6% adherence, while the CON group had 45.7%. The Chi-square test showed no significant difference in RRI incidence across groups (CHI^2^ = 2.958, *p* value = 0.08). A two-way ANOVA showed no significant difference in maximal aerobic speed across groups (*p* = 0.822) or before and after training (*p* = 0.304). Conclusions: This pilot study confirmed the feasibility of this randomized, controlled trial with a needed sample size of 194. However, novice runners had greater attrition rates when starting. Based on those limited data, strengthening activities that replaced running volume did not improve RRI or maximal aerobic speed.

## 1. Introduction

The advantages of running, whether for personal well-being, leisure, or professional pursuits, are widely acknowledged and supported by ample evidence [1,2]. However, scientific research also highlights a significant prevalence of running-related injuries (RRI), ranging from 20% to 80% [3]. Individuals most vulnerable to such injuries are typically inexperienced runners, who may lack the necessary skills to manage running discomfort or modify their training regimen appropriately [4,5].

Running-related injuries (RRI) can be defined as physical complaints that necessitate a modification or the interruption of training or competition [6]. In running, most RRI are musculoskeletal overuse injuries that gradually develop during training [7,8]. Several factors are predictive of RRI, including training errors, such as an increase in the volume, intensity, elevation, or duration of running [9,10,11].

Running stakeholders are eager to discover interventions that can prevent RRI. However, interventions such as foot orthoses [12,13], running shoe prescriptions [14,15,16], specific warm-up and cool-down routines, and stretching and strengthening programs have reported conflicting results, and therefore, no clear recommendations can be made.

It is widely recognized that adding strengthening exercises to a running program can enhance running economy and performance [17,18,19,20]. In team sports such as football or basketball, the incorporation of strength training has been shown to also reduce injury incidence [21]. However, this conclusion cannot be generalized to running. Taddei et al. reported promising results in a randomized, controlled trial with experienced runners who received eight weeks of additional foot core training, resulting in a 2.42 reduction in RRI incidence compared to a non-strengthening group [22]. However, Toresdahl et al. proposed a 12-week marathon training program with the intervention group performing weekly strength exercises, but no difference was observed in RRI incidence compared to a control group [23]. In both studies, strength exercises were added on top of the running program.

Although the addition of strength training to a running program did not appear to prevent RRI, there may be some benefit in reducing the amount of running training and replacing it with strength training. Paavolainen et al. demonstrated that replacing 32% of running with explosive strength training over a nine-week period in elite male runners resulted in greater improvements in maximal aerobic speed and running economy in the intervention group, mainly due to neuromuscular adaptations [20]. RRI incidence was not monitored in this study; however, we can hypothesize that implementing strengthening exercises and reducing running volume may be beneficial in terms of RRI.

The present study had, therefore, multiple objectives. Firstly, it aimed to assess the feasibility of a strengthening-based running program for novice runners, including evaluating the attrition rate dropout potential and calculating the required sample size. Secondly, the study aimed to investigate the effects of the strengthening-based running program on RRI incidence and performance compared to a control group engaging in standard running training.

## 2. Methods

This pilot study was designed as a prospective randomized, controlled trial and followed the CONSORT Checklist of information to include information when reporting a pilot or feasibility trial. It lasted 21 weeks and took place in the Université Catholique de Louvain facilities (Ottignies-Louvain-la-Neuve, Belgium). The recruitment was conducted by word of mouth in the surroundings of the University and local communities. All participants understood French and received information about the protocol and aims of the study. Every included participant provided their signed written consent upon enrollment. The study was approved by the local ethical committee (CEHF, N°: B403201942384). The study was recorded in clinical trial database (NCT05656755).

### 2.1. Participants

Following the recruitment period, the participants were screened for eligibility. They were included if (1) they were between 18 and 30 years old, (2) they matched our definition of novice runners, (3) they had not sustained a musculoskeletal injury in the past six months, and (4) they agreed to subscribe to an online application and to report each session of running training on this platform. A novice runner is defined as someone who has not been running more than once a week for more than six months. The participants were systematically excluded if they did not match the interval of age and were not considered novice runners, i.e., they ran twice or more times a week for more than six months. They were also excluded if they had sustained an injury in the last 6 months or suffered from any musculoskeletal or neurological disorders. Finally, the participants were asked to complete a survey to collect demographical variables such as gender, age, height, weight, regular sport activity, any previous injury, the use of their running shoes, and their contact information. This information was collected on a password-protected file. After completion of the survey, the participants were included for randomization.

### 2.2. Randomization

The participants were randomized with a stratified randomization method (age × gender) using MedCalc^®^ software version 22.016. They were divided into an intervention group (INT) and a control group (CON).

### 2.3. Training Programs

Both groups started a 21-week training program with three sessions per week. Each week, two sessions of one hour were supervised by two investigators, and one mandatory unsupervised session of 30 min was completed by the participants. The supervised sessions took place in the evening on an indoor track, whereas the unsupervised sessions took place at any time on a treadmill or outside. The CON group received a standard running program exclusively composed of supervised running sessions. The training sessions were composed of running (RUN) and recovery (walking or slow running (REC)) periods. The INT group performed strengthening exercises instead of some running volume (static strengthening exercises (SSE) and dynamic strengthening exercises (DSE)). Strength training was performed exclusively with body weight and consisted of exercises such as squats, deadlifts, hip thrusts, and step ups or downs, planks. Estimated time of training in each category for the two groups can be found in Table 1.

### 2.4. Outcomes

#### 2.4.1. Attrition Rate and Adherence

Attrition rate and adherence to the program were recorded throughout the intervention period with the use of an online platform that facilitates communication between the athletes and the coaches (Nolio, Seyssins, France). Attrition rate refers to the percentage of participants who did not complete the training program, whereas adherence refers to how well participants followed the program protocol.

Each participant was instructed to confirm every session that had been performed on the platform. If a participant had not completed his profile on Nolio for one week, a reminder was sent by an online notification. After two weeks of inactivity on Nolio, a phone call was systematically made. A dropout was defined if a participant did not follow three training sessions for any other reason than RRI.

#### 2.4.2. Running Related Injury

RRI was monitored during the follow-up. After each training session, the participants were requested to report any sensation of discomfort. An initial screening was performed by the investigators, and in case of recurrent or crescent pain, an appointment with a physician and/or a physiotherapist of the Belgian Track and Field Federation was made. According to Yamato et al., an RRI was reported if a participant complained about physical issues and interrupted or reduced their training for at least three consecutive sessions or if they consulted a health professional [6].

#### 2.4.3. Running Performance

The maximal aerobic speed (MAS) was chosen to evaluate running performance. For this purpose, the VAM-Eval test was used [24]. A first VAM-Eval test was conducted during the 4th week, and the second was performed during the 21st week. Landmarks were spaced 20 m apart on an indoor athletic track. The starting running speed was set at 8 km·h^−1^, then it was increased every minute by 0.5 km·h^−1^. The test was considered finished if the runner failed to reach the following landmark on time or decided to stop by themself. The last completed stage or half stage, i.e., 30 s into the stage, was considered as the MAS of the participants.

### 2.5. Statistical Analysis

Statistical analysis was performed using MedCalc^®^ Statistical Software version 20.106 (MedCalc Software Ltd., Ostend, Belgium). Descriptive data were collected as mean, standard deviation, minimal, and maximal values. The attrition rate was calculated with the randomized sample at baseline. The adherence was calculated as the number of sessions performed compared to the initial training schedule. The intention-to-treat analysis method was used for the primary outcome, i.e., the incidence of RRI. To determine the distribution of RRI between the INT and the CON, a Chi-squared test was performed. If needed, a Cramer’s V was calculated to demonstrate the effect size of the difference between groups. Then, the Relative Risk (RR) and the Number Need-to Treat (NNT) in regard to RRI were all calculated following the methods described by McCoy et al. [25]. A two-way, repeated ANOVA (Group × Time) was executed to compare MAS performance. The Alpha of significance was set at 0.05.

## 3. Results

**Figure 1:**
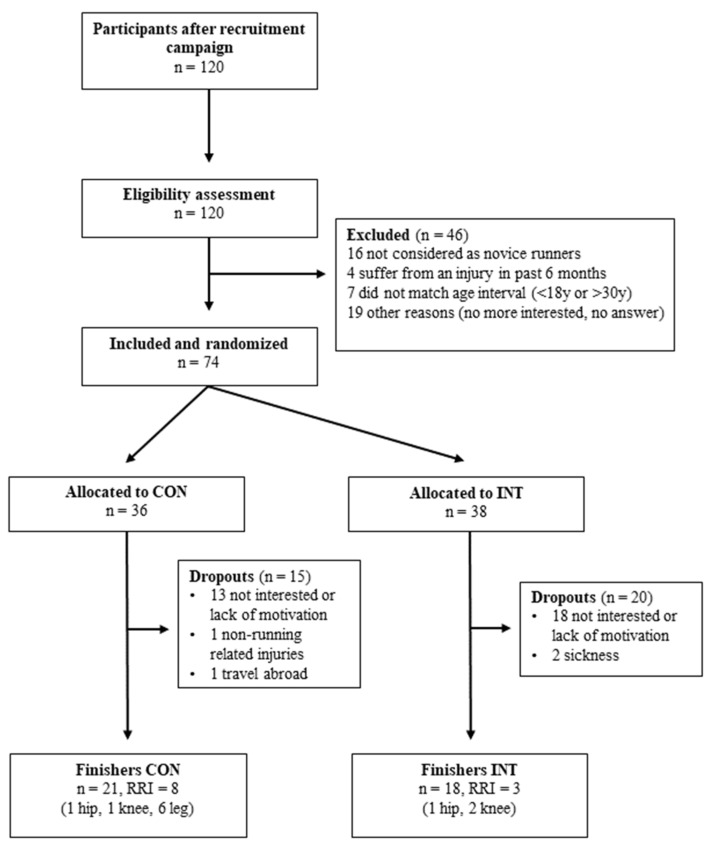
Flow diagram of participants. CON: control group; INT: intervention group.

A total of 120 individuals responded to the study recruitment campaign, with 46 that did not match the eligibility criteria. Therefore, 74 novice runners were randomized into the two groups, with 38 in the INT group and 36 in the CON group (Figure 1). The baseline descriptive characteristics can be found in Table 2.

### 3.1. Attrition Rate and Adherence

The attrition rate was 52.6% in the INT group and 41.7% in the CON group (20 and 15 dropouts, respectively). Thirty-one participants stopped their participation due to a lack of interest or motivation; one had a non-RRI, one traveled abroad, and two were ill. The adherence rates to the training programs were 45.7% in the CON group and 56.6% in the INT group, respectively.

### 3.2. Running Related Injuries

Eleven runners out of the thirty-nine participants who completed the study reported having an RRI. Eight runners reported an RRI in the CON group (38%), of which one non-identified glute muscle pain, one identified patellofemoral pain syndrome, one identified ilio-tibial band syndrome, two identified medial tibial stress syndromes (MTSS), and three identified non-identified calf or tibial pain. Three runners reported an RRI in the INT (16.6%), of which one had greater trochanteric pain syndrome, and two had patellar tendinopathies.

The Chi-squared test failed to demonstrate a significant difference in RRI incidence between CON and INT groups (χ^2^ = 2.958, *p* value = 0.08). The implementation of the strengthening program while reducing the total running volume in the INT group compared to the CON did not report a significant reduction in the risk of injuries (RR = 0.355, 95% confidence interval (95%CI) of (0.102 to 1.235), *p* value = 0.1; z = 1.628). The NNT concerning the INT group was seven runners.

### 3.3. MAS

The two-way, repeated measure ANOVA did not demonstrate an interaction (Group × Time) for MAS with a *p* value of 0.119. There was no GROUP (*p* value = 0.822) or TIME (*p* value = 0.304) effect.

## 4. Discussion

To our knowledge, this is the first study to investigate the effect of replacing running volume by strengthening exercises in novice runners on RRI and running performance. Our sample, comprised of university students, exhibited a high attrition rate and relatively low adherence to the training programs. Based on our limited sample results, both groups reported a similar risk of injuries and no increase in MAS.

### 4.1. Attrition Rate and Adherence

The high rate of attrition and the low rate of adherence should invite the reader to consider those results with caution. However, the high attrition rate was previously reported in a novice runner population. Some authors reported a 47% dropout rate in novice runners [26], while another study reported a 75% dropout rate in an obese novice runner population [27]. It is worth noting that our sample consisted entirely of university students who were recruited at the start of the academic year. These individuals may not have fully understood the demands of three weekly running sessions over a 21-week program. Furthermore, the student population is highly influenced by contextual factors such as holidays or exam sessions that could impair their participation in social events such as sports activities and, more precisely, a new one that could not be habitual yet. In addition, adherence and session completion were recorded through an online application. Some participants could have omitted or did not have the rigor to register their training, which may lead to an overestimation of uncompleted sessions.

We thought that the inclusion of strength training could provide greater variation in training sessions and limit monotony. This is not confirmed, as both groups reported similar levels of attrition and adherence. Motivational factors of running practice in novice runners were mostly influenced by schedule constrain, climate conditions, social support, or previous levels of physical activity [28].

### 4.2. Running-Related Injuries

While the two groups did not report significant differences in RRI over the 21 weeks of training, there seems to be a clinical tendency that replacing running volume with strengthening exercises might reduce the incidence of RRI. To date, only foot-core training has shown a positive impact on the prevention of RRI [29], while other strengthening methods did not show any significant results [23,30]. One explanation is that similar running volume and duration rather than strengthening modalities could be relevant considering RRI incidence. In the previous studies, strengthening was added to the running program resulting in an increase in the total workload. We might suggest that this increase may cancel the benefit of strengthening. That is why we followed the recommendations of some coaches to replace the running workload by strengthening exercises [31]. This reduction of volume is thought to induce less repetitive mechanical stress on untrained musculoskeletal tissue in novice runners and reduce RRI.

While Lauersen et al. showed, in their systematic review and meta-analysis, that loaded strength training was helpful in preventing sports overuse injuries in soccer, basketball, and football by approximately 50% [21], we decided to employ bodyweight muscular strength training in our study. Explosive sports such as sprinting or soccer that are related to short bursts of speed and acceleration could be more respondent to muscle quality and function such as force or power. In contrast, bodyweight muscle strength training was thought to be meaningful in inducing variable workload on the lower limbs for endurance runners, specifically novice ones.

Eight of the eleven reported injuries occurred in the first seven weeks of training. A pilot study by Baltich et al. on novice runners found the highest incidence of RRI in the first eight weeks of the four-month training program [26]. In contrast, Kluitenberg et al. reported a higher rate of RRI during the second and third weeks of their six-week supervised “Start to Run” program for novice runners [32]. This emphasizes the criticality of early training sessions for novice runners. Coaches, strength and conditioning coaches, or healthcare practitioners should prioritize investing time and attention during the early training periods.

### 4.3. Performance

Many studies have already proved the benefits of adding heavy or plyometric strength training programs on performance and running economy [17,18,19,23,33,34], untrained endurance runners should preferably follow a maximal-strength-oriented program to enhance maximal force, power, and reactive-strength capabilities, which will lead to an improvement in running economy. This differs from well-trained endurance runners with high-force capabilities who should focus on specific explosive and reactive-strength training to improve performance [24]. A replacement of a part of running volume by explosive-type strengthening (sprints, jumps, leg press, and knee extensors–flexors exercises) also showed improvements in terms of 5 km running performance and aerobic power in elite orienteering runners [20]. This could explain the non-efficacy of our strengthening program, as it was general and more focused on endurance strength development.

Therefore, novice runners could benefit from load muscle strengthening. Using a 1RM-based training program, rather than bodyweight-based exercises, could lead to greater improvements in maximal force, strength endurance, and power. Moreover, 1RM-based programs produce better results than bodyweight-based programs in terms of strength gains and muscular endurance [25] and should be studied in the future.

In experienced runners, some studies found an existing correlation between running workload, specifically running volume, and running performance [35,36]. The research suggests that factors such as training volume and personal best time in marathons are associated with performance in male 100 km ultrarunners [36]. Similarly, the volume of training and the longest endurance runs have been found to be related to performance and running injuries in marathon training [35]. Consequently, it can be hypothesized that reducing running volume may hinder the development of aerobic performance, such as maximal aerobic speed. However, this particular outcome was not investigated in the present study and, therefore, cannot be confirmed. Additionally, caution should be exercised when considering the external validity of these findings. The study did not demonstrate improvements in the maximal aerobic speed for either the intervention or control group. Several factors could contribute to this outcome. Firstly, the participants may not have been as inexperienced as they claimed during enrollment, or they could have been engaged in other forms of physical activity. Secondly, both running programs may have been primarily designed for injury prevention rather than performance enhancement. Thus, for future research, it would be beneficial to include another control group following a performance running program. To summarize, the impact of strength training on mitigating the decline in aerobic capacity caused by reduced running volume in novice runners remains unclear and warrants further investigation.

### 4.4. Limitations

The present study is not without limitations. First, the high rate of attrition made it imperative to proceed with caution when interpreting the findings of this study. Another limitation is the non-measurement of muscle function, such as muscle maximal force, power, or endurance. The strengthening program was not confirmed as efficient in increasing muscle force and, therefore, could provide unclear results. In addition, while recruiting only novice runners, the present study did not report any baseline value of physical activity and sport level. Furthermore, no gender analysis was performed. This could lead to more heterogenicity of the sample. Finally, the study did not provide any follow-up after the 21 weeks of sessions. These limitations make the present results unfit for external validity, and researchers are invited to perform further studies.

### 4.5. Perspectives

Based on the proportion of RRI in our sample, we would need 194 participants randomized into two groups to observe a significant RR (Type I error: 0.05, Type II error: 0.2). As it could be potentially complicated, the need to decrease attrition and increase adherence is needed. Therefore, we postulate four recommendations to optimize attrition and adherence. We believe that a 12-week program would be sufficient to observe a difference in RRI incidence as novice runners tend to report RRI in their first trimester of practice. Taddei et al. attested that an 8-week foot-core program could diminish the risk of RRI by 2.42 times in a 12 month follow-up period in recreative runners [29]. On the other hand, the session should be practiced in groups and with supervision to enhance participation and adherence. In addition, the follow-up of RRI or dropout should be recorded within the following year at least. Future studies should avoid taking only university students and avoid holiday seasons. Finally, it was reported that a clear objective could help adherence [37]. The objective of a race could benefit and motivate novice runners to end the training program.

## 5. Conclusions

Based on the limited results of this pilot study, there was no reduction of RRI in novice runners that reduced running milage and implemented a bodyweight strengthening program. The present study provided the insight needed to prepare a more robust clinical study to permit relevant results to guide sports and health practitioners.

## Figures and Tables

**Table 1:** Time in minutes planned per person in each training category.

Category	CON	INT
RUN	2065	1606
REC	329	207
SSE	0	214
DSE	8	387
Total training time	2402	2414

RUN: running; REC: recovery; SSE: static strengthening exercises; DSE: dynamic strengthening exercises; CON: control group with only running; INT: intervention group with strengthening and running training.

**Table 2:** Baseline characteristics of runners.

		CON (n = 36)	INT (n = 38)
Gender	Male	18	18
	Female	18	20
Age (years)		20.8 ± 2.6	20.9 ± 2.2
Height (cm)		173.5 ± 9.6	173.5 ± 7.8
Weight (kg)		69.2 ± 12.2	67.1 ± 9.3
BMI		23 ± 3.1	22.4 ± 2.9

BMI: body mass index.

## Data Availability

Data are unavailable due to privacy policies.
